# Relationship between Thoroughbred workloads in racing and the fatigue life of equine subchondral bone

**DOI:** 10.1038/s41598-022-14274-y

**Published:** 2022-07-07

**Authors:** Ashleigh V. Morrice-West, Peta L. Hitchens, Elizabeth A. Walmsley, Kate Tasker, Ser Lin Lim, Ariel D. Smith, R. Chris Whitton

**Affiliations:** Melbourne Veterinary School, Equine Centre, University of Melbourne, 250 Princes Hwy, Werribee, VIC 3030 Australia

**Keywords:** Computational models, Statistical methods, Computational biology and bioinformatics

## Abstract

Fatigue life (FL) is the number of cycles of load sustained by a material before failure, and is dependent on the load magnitude. For athletes, ‘cycles’ translates to number of strides, with load proportional to speed. To improve previous investigations estimating workload from distance, we used speed (m/s, x) per stride collected using 5 Hz GPS/800 Hz accelerometer sensors as a proxy for limb load to investigate factors associated with FL in a Thoroughbred race start model over 25,234 race starts, using a combination of mathematical and regression modelling. Fore-limb vertical force (NKg^-1^) was estimated using a published equation: Vertical force = 2.778 + 2.1376x − 0.0535x^2^. Joint load (σ) was estimated based on the vertical force, scaled according to the maximum speed and defined experimental loads for the expected variation in load distribution across a joint surface (54-90 MPa). Percentage FL (%FL) was estimated using a published equation for cycles to failure (N_f_) summed across each race start: N_f_ = 10^(σ-134.2)/−14.1.^ Multivariable mixed-effects linear regression models were generated on %FL, adjusting for horse-level clustering, presented as coefficients; 95%CI. Scaled to the highest joint load, individual starts accrued a mean of 9.34%FL (sd. 1.64). Older age (coef. 0.03; 0.002–0.04), longer race-distances (non-linear power transformed), and firmer track surfaces (ref. Heavy 10: Good 3 coef. 2.37; 2.26–2.48) were associated with greater %FL, and males accrued less than females (*p* < 0.01). Most variables associated with %FL are reported risk factors for injury. Monitoring strides in racehorses may therefore allow identification of horses at risk, enabling early detection of injury.

## Introduction

Numerous studies have investigated associations between workload and bone injuries in Thoroughbred racehorses, but are limited by the practicality of measuring workload itself^1–8^. Most have relied on speed and distance travelled both in training and racing to quantify workload, and often use estimates of speed which lack precision^9, 10^. For human athlete monitoring, GPS data with distance travelled categorised according to speeds achieved have been used^11, 12^. GPS data for speed and distance has also been used to estimate workloads in a limited number of equine studies but for small sample sizes under non-competition conditions ^13–16^.

Racehorse bone injury is most commonly a result of bone fatigue. This process leads to the degradation of the bone’s overall integrity generating microdamage and microcracks which can propagate, resulting in bone failure^17–20^. The fatigue life of a material is the number of cycles of a defined load able to be incurred before partial or complete failure^17, 21^. In racehorses fatigue life translates to the number of strides accrued before localised failure or fracture occurs. The fatigue life of bone is dependent on the magnitude of load via an inverse exponential relationship and has been investigated in cortical, trabecular and subchondral bone^22–24^. It is not yet possible to measure load in limbs while horses are competing, but load on the equine limb is proportional to speed^25^, therefore, as galloping speed increases, a substantially lower number of stride cycles are required to induce injury^22^.

A limited number of studies have investigated the loading capacity on equine bone, with subchondral bone failing the earliest under repeated compressive loading at only 4000 cycles^22^. Musculoskeletal injuries occur at various locations in Thoroughbred racehorses, but the metacarpo(/metatarso)-phalangeal (fetlock) joints, and in particular, the subchondral bone of the distal palmar aspect of the third metacarpal (/tarsal) condyles are most frequently affected^26–28^. Of all the distal limb, this joint is subjected to the highest stress, with vertical load on the limb amplified at the joint surface by the fetlock moment arm^29^. The repeated high joint surface loads result in fatigue failure of subchondral bone, with damage either spreading transversely across the joint surface (palmar osteochondral disease) or propagating proximally as parasagittal fractures^26–29^. Given the high loads incurred on the fetlock joint, and the frequency of condylar disease/fracture, the condylar subchondral bone was the focus of this investigation.

Epidemiological studies have provided substantive evidence that horse-level factors including older age and male sex, and race-level factors including firmer turf track surfaces, longer race distances, higher classed races and larger field sizes are associated with catastrophic musculoskeletal injury (CMI)^6^. We have previously described a large variation in the stride characteristics of a cohort of Australian Thoroughbreds during racing^30^. By quantifying the number of cycles, analogous to strides, incurred during racing we aimed to determine the loading history of equine bones in vivo. The variation in stride parameters associated with horse- and race-level factors is likely to result in variation in the degree of bone damage accrued and therefore the propensity for injury. We aimed to use published fatigue equations to assess the effect of racing workloads as determined by distance and speed on equine subchondral bone fatigue. Based on the large inter-horse variation in stride parameters and known risk factors for injury, we hypothesised that a higher percentage of bone fatigue life would be accumulated by horses (1) competing in longer distance races; (2) on firmer track surfaces and synthetic surfaces compared to heavier turf tracks; and (3) in higher classed races; and that (4) some horses will accumulate greater fatigue than others based on their innate stride characteristics.

## Materials and methods

### Data sources

Retrospective speed and stride data (stride length, duration) from Thoroughbred racehorses in n = 33,459 race starts between 11 of January 2011 and 21 of August 2016 were sourced from Tasracing and the product and software manufacturer (StrideMaster™; Thoroughbred Ratings Pty Ltd, Romsey, Victoria, Australia)^30^. Data for the present study were provided as SQLite files by individual stride, collected from 5 Hz positional recording and 800 Hz accelerometer recordings via sensors mounted on the saddle cloth of each horse in every race start, and smoothed through StrideMaster™ proprietary algorithms. Individual stride data were then matched with race result data from the official racing repository (Racing Australia Ltd) for finishing position, weight carried, track type and surface, race class and distance, and previous number of starts from our previous study^30^. The database was cleaned, and observations sequentially excluded as previously described in detail ^30^. Briefly, duplicates, starts outside the study period, non-starters and recording errors (missing speed and stride records), including horses that pulled up, lost a rider, fell or were injured or disqualified were excluded (n = 7,314 starts). An additional n = 634 starts were unable to be matched to official racing repository records (n = 25,511 matched race starts). Race-starts with biologically implausible 200 m sectional averages (n = 252 starts) and an additional n = 25 starts assessed at the individual stride level were excluded according to the exclusion criteria: speeds < 12.57 m/s or > 21 m/s, stride lengths < 5.3 m or > 9.2 m and stride durations < 0.37 s or > 0.49 s. Resultingly, n = 25,234 race starts were available for analysis, conducted by n = 2,676 individual horses.

Horse age was treated as a continuous variable in years. Weight carried was defined according to the Australian Rules of Racing to include the combined weight of the rider, all items of clothing worn by the rider (except helmet, face protection and gloves), saddle and any additional attached gear, lead bag and associated packing (Australian Rules of Racing, As at 1 March 2019, AR184), recorded per kg in 0.5 kg increments (categorised per 2.5 kg for descriptive data and scaled per 10 kg for regression modelling). Track surfaces were classified as synthetic or turf, with turf tracks rated from firmest to most water-logged in ordinal categories (2–10). Race classes were categorised as: (1) Maiden and Class 1 races (horses that had won ≤ 1 race); (2) Class 2–5 (where starters have won ≤ 2–5 races respectively); (3) Benchmark (BM) and Handicap (HCP) races (restricted races in which horses are assigned weights based on their rating); (4) Open races (available to all horses); and (5) Listed and Group races (highest classed races). Race distance was recorded per metre (m) and scaled per 100 m to enable interpretable coefficients in regression modelling^30^.

### Mathematical modelling

Vertical force (NKg^-1^, normalised to the combined horse body weight, rider and equipment weight) exerted on the fore-limb at each stride was estimated according to the formula by Witte, et al.^25^, Eq. (1), based on speed (m/s; x) given that stance duration was not available, adjusting the sign of the b_0_ (constant) term to reflect the quadratic relationship displayed graphically in that paper:

1 $$Estimated \, vertical \, force = \, 2.778 \, + \, 2.1376x \, - \, 0.0535x^{2}$$

Equation (1) resulted in a maximum vertical force (at maximum speed of 20.99 m/s) of 24.13 NKg^-1^. To scale to the load at a distal limb joint for the calculated vertical force at each stride, we referred to previously published ex *vivo* joint loads at the metacarpal condyle, which has been reported to represent 49 to 80% of yield stress (i.e. the singular stress required to induce failure)^22, 31^. Because load will vary across a joint surface^32^, we ran a sensitivity analysis where estimated joint loads were calculated based on maximally defined experimental loads of 90, 78, 66 and 54 MPa as per Eq. (2) ^22^. Joint load (MPa; σ) was estimated as the product of the conversion factor and the estimated vertical force from Eq. (1).

2 $$Conversion \, factor \, = \, \left( {experimental \, load} \right)/{24}.{13}NKg^{ - 1}$$

3 $${ }\sigma \, = \, \left( {Conversion \, factor} \right) \cdot \left( {estimated \, vertical \, force} \right)$$

The number of cycles to failure (N_f_) was calculated according to the fatigue life curve for equine metacarpal subchondral bone with known load, Eqs. (4) and (5) ^22^.

4 $$\sigma = 134.2 - 14.1 \cdot log10\left( {N_{f} } \right)$$

5 $$N_{f} { } = 10^{{\left( {{\upsigma } - 134.2} \right)/{-}14.1}} { }$$

The proportion of fatigue life accrued during each recording (i.e. each stride) for each scaled load was calculated as the quotient of the number of strides (one) and the number of cycles to failure (N_f_), Eq. (6). The sum of the proportions of fatigue life for each recording were then calculated for each race start at each scaled load, and converted to a percentage to generate the race percentage fatigue life (FL_% race_), Eq. (7).

6 $$FL_{stride} { } = { }1/N_{f}$$

7 $$FL_{\% race} = { }sum\left( {FL_{stride} } \right) \cdot 100$$

### Statistical analysis

Data analysis was performed using Stata (Stata Version 15.0 StataCorp, College Station, TX: StataCorp LP). Data were assessed for normality via histograms and Shapiro–Wilk tests and accordingly presented as means (standard deviation; sd.) and ranges. Data were collapsed by race start, and summary statistics for the race percentage FL at each scaled load (90, 78, 66, 54 MPa) were generated, stratified by race factors of class, distance, and track surface.

Using the collapsed data to assess the effect of horse- and race-level factors on the percentage fatigue life accrued over each race start, univariable linear regression models were generated using the highest scaled load (90 MPa), adjusting for horse-level random effects to account for multiple starts within horse. To determine whether linear regression on the outcome variable percentage fatigue life was appropriate, we assessed predicted values of the model to ensure they did not fall outside the bounds of 0 and 100%^33^.

Study factors assessed included horse sex and age, weight carried, finishing position, track type and rating, race class, and race distance. Univariable model fit was assessed using both categorical and continuous versions of the variables weight carried and race distance. For both variables residual normality and information criterions were improved with the continuous variables therefore the latter were used for subsequent multivariable modelling. Continuous predictor variables were assessed for linearity of association with the outcome variable, with potential transformations assessed according to improvement in AIC and BIC values, model specification tests (linktest) and normality of residuals assessed via histograms. A Box-Tidwell (power) transformation was applied to the predictor race distance (per 100 m), with a new variable generated for use in further modelling according to Eq. (8) ^34^.

8 $$Race\, distance_{ Transformed} = \left( {\frac{{{\text{Race distance }}\left( {\text{m}} \right)}}{100}} \right)^{0.353}$$

All variables *p* < 0.2 were entered in multivariable models, then retained if *p* < 0.05 following backwards stepwise elimination. Sequential model fit was assessed by minimisation of AIC and BIC values. Potential two-way interaction terms were evaluated, with statistically significant terms assessed graphically. Model diagnostics were conducted for assessment of normality of residuals and graphical comparison of predicted and actual values to assess goodness of fit.

### Ethics approval

The Animal Ethics Committee at the University of Melbourne Faculty of Veterinary and Agricultural Science gave an exemption for formal ethics approval due to the studies use of existing collections of data

### Consent to participate

Data collection is required by the racing authority (Tasracing). Tasracing representatives gave consent for this study.

### Consent for publication

All authors gave approval for publication.

## Results

**Figure 1:**
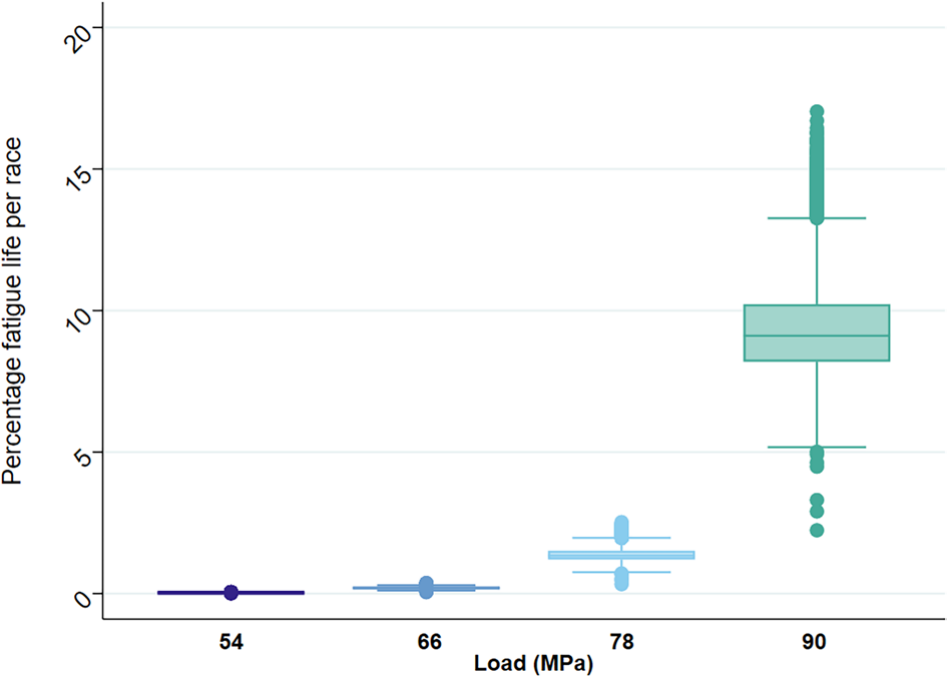
Box-plot showing the percentage fatigue life accrued in 25,234 Thoroughbred race starts in Tasmania, Australia when scaled to pre-determined experimental joint loads.

Descriptive data for the horse- and race-level factors analysed for n = 25,234 race starts are presented in Table 1. Horses were a mean of 4.83 (sd. 1.43) years of age, with 43.52% of starts by female horses (n = 10,983), 54.43% by geldings (n = 13,735) and 2.04% by entire males (n = 516). The estimated percentage of fatigue life accumulated over each race start stratified by race distance, track surface type and rating are presented in Table 1 as mean ± sd. The sensitivity analysis to account for variation in force distribution across a joint surface demonstrated the exponential relationship between the joint surface load and percentage fatigue life (Eq. 5) accrued over each race start (Fig. 1).

**Table 1:** Estimated percentage of bone fatigue life accrued over n = 25,234 Thoroughbred race starts in Tasmania, Australia as means (sd.) based on speed and stride data obtained by GPS and accelerometer. Results are stratified by race distance of race and track surface type and rating (firmest to softest). Loads were estimated using varying pressure across a joint surface according to pre-determined experimental joint loads. For categorical variables, number of starts (N) and percentage of starts are presented. Continuous variables are presented as mean (sd.), stratified by experimental load indicating variation in race percentage fatigue life.

Scaled load (MPa)		54	66	78	90
		Race percentage fatigue life accrued			
All starts		0.03 (0.01)	0.20 (0.04)	1.38 (0.25)	9.34 (1.64)
	N(%) / mean (sd.)				
**Race distance (m)**	1,372.66 (312.73)				
≤ 1200	7094 (28.11%)	0.02 (0.002)	0.17 (0.01)	1.14 (0.10)	7.8 (0.70)
> 1200—≤ 1600	11,704 (46.38%)	0.03 (0.002)	0.20 (0.02)	1.35 (0.11)	9.15 (0.82)
> 1600—≤ 2000	4286 (16.99%)	0.04 (0.002)	0.24 (0.02)	1.6 (0.13)	10.71 (0.92)
> 2000—≤ 2400	1994 (7.9%)	0.04 (0.002)	0.29 (0.02)	1.9 (0.14)	12.58 (1.07)
> 2400	156 (0.62%)	0.05 (0.003)	0.33 (0.02)	2.16 (0.15)	14.32 (1.16)
**Track type and rating**					
Synthetic	6,966 (27.61%)	0.03 (0.01)	0.20 (0.04)	1.32 (0.24)	8.96 (1.55)
Turf					
Firm 2	20 (0.08%)	0.02 (0.001)	0.17 (0.01)	1.16 (0.07)	7.95 (0.47)
Good 3	1,448 (5.74%)	0.03 (0.01)	0.22 (0.04)	1.48 (0.26)	10.03 (1.73)
Good 4	7,324 (29.02%)	0.03 (0.01)	0.21 (0.04)	1.46 (0.24)	9.89 (1.59)
Soft 5	4,707 (18.65%)	0.03 (0.01)	0.21 (0.04)	1.41 (0.25)	9.51 (1.62)
Soft 6	1,355 (5.37%)	0.03 (0.01)	0.20 (0.03)	1.33 (0.21)	8.91 (1.34)
Soft 7	1,215 (4.81%)	0.03 (0.01)	0.20 (0.04)	1.33 (0.24)	8.86 (1.55)
Heavy 8	1,606 (6.36%)	0.03 (0.005)	0.19 (0.03)	1.29 (0.20)	8.55 (1.29)
Heavy 9	399 (1.58%)	0.03 (0.01)	0.19 (0.03)	1.24 (0.21)	8.21 (1.33)
Heavy 10	194 (0.77%)	0.03 (0.005)	0.18 (0.03)	1.14 (0.19)	7.42 (1.24)
**Race class**					
Maiden/Class 1	12,933 (51.25%)	0.03 (0.01)	0.20 (0.03)	1.32 (0.22)	8.93 (1.45)
Class 2–5	2,689 (10.66%)	0.03 (0.003)	0.19 (0.02)	1.30 (0.13)	8.87 (0.92)
Restricted (HCP/BM)	8,186 (32.44%)	0.03 (0.01)	0.22 (0.04)	1.48 (0.26)	9.98 (1.71)
Open	573 (2.27%)	0.03 (0.01)	0.20 (0.04)	1.36 (0.27)	9.28 (1.76)
Listed /Group	853 (4.48%)	0.04 (0.01)	0.26 (0.05)	1.72 (0.35)	11.58 (2.23)
**Weight carried (kg)**	55.78 (2.05)				
50- 52	1,539 (6.1%)	0.03 (0.01)	0.21 (0.04)	1.39 (0.25)	9.39 (1.64)
52.5- 54.5	6,244 (24.74%)	0.03 (0.01)	0.21 (0.04)	1.39 (0.26)	9.40 (1.71)
55- 57	11,866 (47.02%)	0.03 (0.01)	0.20 (0.04)	1.38 (0.25)	9.29 (1.64)
57.5- 59.5	5,317 (21.07%)	0.03 (0.01)	0.20 (0.04)	1.38 (0.23)	9.32 (1.55)
≥ 60	268 (1.06%)	0.03 (0.01)	0.22 (0.04)	1.45 (0.27)	9.85 (1.73)

**Figure 2:**
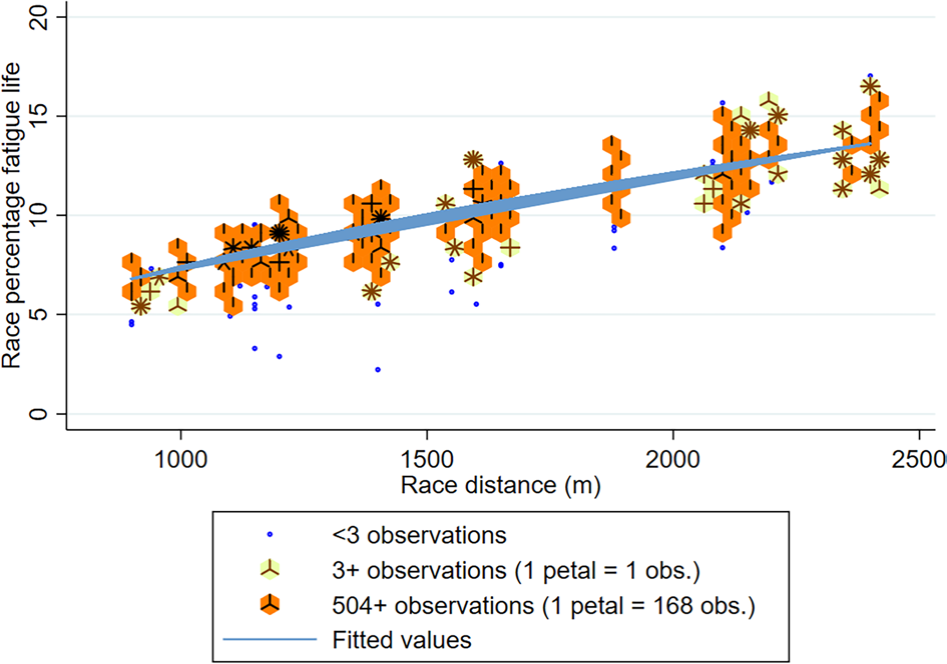
Scatter density plot of race distance to the percentage of fatigue life accrued per race with Box-Tidwell power transformed fitted linear regression line from the final multivariable model (transformed race distance = race distance per 100 m^0.353^). Petals of shaded areas (“sunflowers”) represent the number of observations, where the number of observations increase from blue (< 3 observations) to shaded sunflowers of yellow (3 to 503 observations) to orange (504 + observations). Overlapping lines (indicating greater number of overlapping petals) equate to the highest density regions within each sunflower.

**Figure 3:**
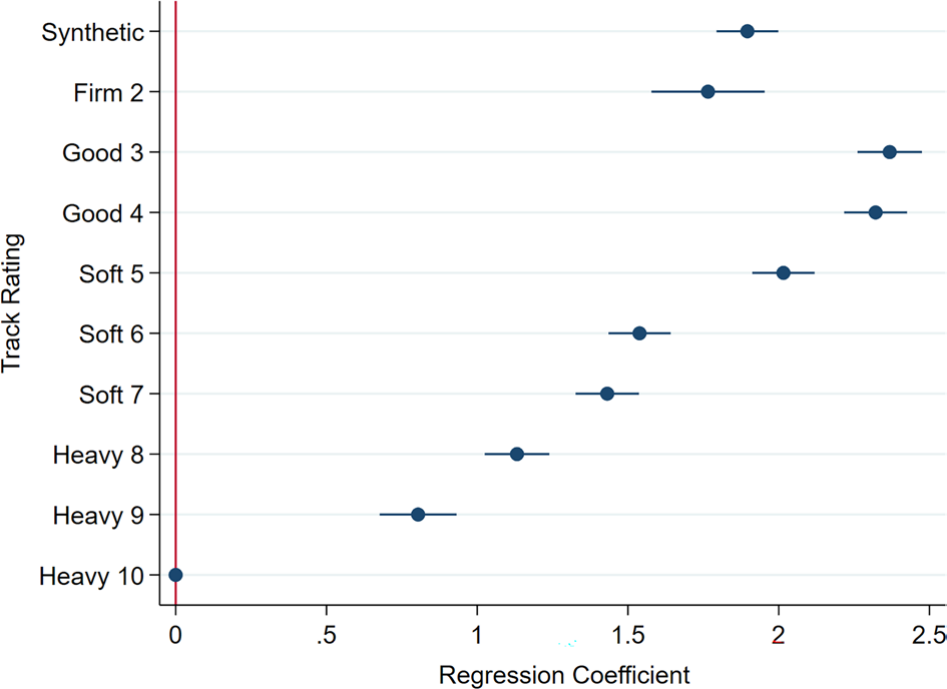
Coefficient plot (regression coefficients and associated 95% confidence intervals) showing the proportional effect of track surface type and rating (synthetic vs turf tracks rated from firmest to softest) on the percentage of fatigue life accrued over Thoroughbred race starts in Tasmania, Australia, according to estimated joint loads scaled to a maximum of 90 MPa.

**Figure 4:**
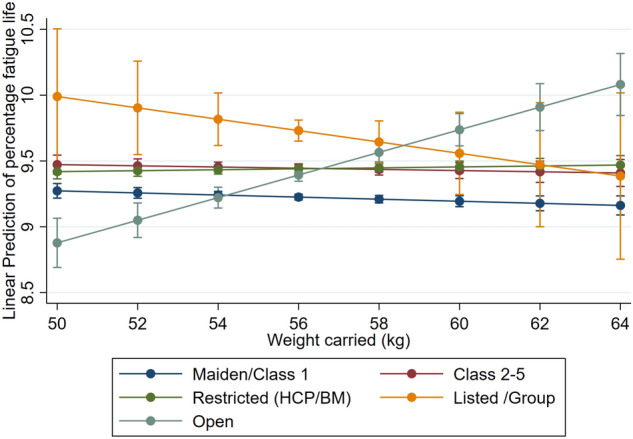
The interaction between weight carried and race class on the percentage of fatigue life accrued over Thoroughbred race starts in Tasmania, Australia in multivariable linear regression modelling, according to estimated joint loads scaled to a maximum of 90 MPa. *HCP/BM = Handicap/Benchmark races.

Univariable regression results for the horse- and race-level factors predicting the percentage fatigue life accrued per race start scaled to 90 MPa joint force loading are presented in Supplementary Table S1. In multivariable analysis, horse-level factors of age and sex were associated with the percentage of fatigue life accrued, with more fatigue life accrued per race as age of horse increased, and lower proportion of fatigue life accrued for male horses than females, Table 2. Greater race distances were associated with greater percentage of fatigue life accumulated in a non-linear fashion (*p* < 0.001), therefore doubling the distance raced did not equate to double the percentage fatigue life accrued, Fig. 2. Firmer (Good rated) track surfaces were associated with greater fatigue life accrued (*p* < 0.001), Fig. 3. The estimated fatigue life accrual for synthetic tracks was between a turf condition of soft 5 and 6, Fig. 3 (statistically different from all turf condition categories; *p* < 0.001). Better finishing positions were associated with a greater percentage of fatigue life accumulated (*p* < 0.001). An interaction was present between weight carried and race class, whereby lower class and restricted races maintained the percentage of fatigue life accrued as weight carried increased. There was some reduction in race percentage fatigue life for high class races (Listed/Group races) as weight increased, but for open class races (races with no special conditions or restrictions), the greater the weight carried the higher the race percentage fatigue life (Fig. 4), however there was only a small number of open class race starters carrying 60 kgs or more (n = 19 of the total 238 race starts carrying this weight). This class of race is available to horses of all abilities (i.e. races with the greatest variation in horse ability).

**Table 2:** Multivariable linear regression results showing the association between horse and race-level factors on the percentage of fatigue life accrued over Thoroughbred race starts in Tasmania, Australia, according to estimated joint loads scaled to a maximum of 90 MPa. Results are presented as coefficients and associated 95% Confidence Intervals. The variance at the individual horse level (random effect term) is presented as standard deviation and Intraclass Correlation Coefficient (ICC).

Race percentage fatigue life	Coef	[95% Conf Interval]		*p*-value
**Fixed effects**				
** Horse factors**				
Sex				
Female	(reference)			
Gelding	− 0.159	− 0.197	− 0.121	< 0.001
Colt/Stallion	− 0.132	− 0.233	− 0.032	0.001
*wald p-value*				< *0.001*
Age (years)	0.028	0.015	0.041	< 0.001
Finishing position	− 0.045	− 0.048	− 0.042	< 0.001
Weight carried (per 10 kg)	− 0.079	− 0.165	0.006	0.070
** Race Factors**				
Race distance per 100 m ^a^	7.353	7.263	7.444	< 0.001
Track type & rating				
Synthetic	1.896	1.794	1.999	< 0.001
Firm 2	1.766	1.578	1.953	< 0.001
Good 3	2.368	2.261	2.475	< 0.001
Good 4	2.321	2.217	2.426	< 0.001
Soft 5	2.016	1.912	2.119	< 0.001
Soft 6	1.539	1.435	1.642	< 0.001
Soft 7	1.431	1.326	1.537	< 0.001
Heavy 8	1.132	1.025	1.239	< 0.001
Heavy 9	0.804	0.676	0.932	< 0.001
Heavy 10	(reference)			
*wald p-value*				< *0.001*
Race Class				
Maiden/Class 1	(reference)			
Class 2–5	0.030	− 0.739	0.799	0.939
Restricted (HCP/BM)	− 0.427	− 1.080	0.225	0.199
Listed /Group	2.480	− 2.104	7.065	0.289
Open	− 5.087	− 6.791	− 3.383	< 0.001
*wald p-value*				< *0.001*
**Interaction effects**				
Race Class: Weight Carried (per 10 kg)				
Maiden/Class 1: Weight Carried	(reference)			
Class 2–5: Weight Carried	0.034	− 0.104	0.172	0.631
Restricted (HCP/BM): Weight Carried	0.115	− 0.002	0.232	0.054
Listed /Group: Weight Carried	− 0.353	− 1.167	0.462	0.396
Open: Weight Carried	0.938	0.635	1.242	< 0.001
*wald p-value*				< *0.001*
Intercept	− 0.386	− 0.871	0.098	0.118
**Random Effects**	Sd. (ICC)			
Horse	1.300 (0.370)			

^a^Race distance (scaled per 100 m) transformed according to a Box-Tidwell transformation: transformed race distance = race distance^0.353^.

The Intraclass Correlation Coefficient for the intra-horse level variation was 0.37 (95% CI 0.35, 0.41), that is, 37% of the total unexplained variation in percentage fatigue life accrued over each race start was attributable to unmeasured horse-level effects.

## Discussion

Using individual stride characteristics recorded during racing in a large population of horses, we aimed to assess variation in the proportion of subchondral bone fatigue life consumed per race. Models produced for different joint surface loads demonstrated that the proportion of fatigue life per race increased exponentially based on the magnitude of the scaled load, but there was substantial variation between horses. Moreover, unmeasured horse effects (i.e. innate qualities of individual horses) accounted for a large proportion of the variance in fatigue life consumed between race starts. Older horses, females, better finishing positions, firmer track surfaces, and longer race distances were estimated to accrue a greater percentage of fatigue life over each race start. Synthetic surfaces were associated with accrued fatigue life that was more similar to soft-rated than good-rated surfaces. The effect of race class on fatigue accumulation was dependent on the weight carried.

In assessing race-day workload in equine athletes, we included the knowledge that bone fatigue injuries are typically the result of cumulative loading cycles at areas of high stress. Comparatively recent studies assessing training and competition workloads in human athletes using GPS data have categorised speed ranges to quantify the distance travelled by the athletes as a proxy for ‘load’^11, 12^. Because cumulative fatigue damage is the most common mechanism for injury, we included a measure of bone fatigue rather than just distance and speed. As we were unable to measure load on bone itself, we used horse speed for each stride as a proxy for the load incurred. Loads were scaled based on joint surface loads expected to be incurred during high-speed galloping. Maximal joint surface pressures in vivo are yet to be determined owing to the difficulty in making such measurements in the live animal, therefore we modelled a range of loads^22, 32^. This method of using speed and cycles to calculate workload based on the fatigue process, whilst subject to limitation, improves upon previous quantitative estimates using just speed and distance.

When loads were scaled to 54 MPa the mean race start fatigue accumulation was only 0.03% of total fatigue life. However, when load was increased by two-thirds, the mean race start fatigue accumulation increased to 9.34% of fatigue life, a greater than 300-fold increase. The exponential relationship between the estimated fatigue accrued for each race start and the magnitudes of the potential loading emphasises the importance of the magnitude of load for injury risk^35^. Limb loading is directly proportional to speed, therefore greater bone damage would be expected to occur with greater race speeds^22, 29, 32^. These relationships explain why cycles of loading over distances raced at high speed have a greater impact on injury than distances at lower speed^8^. Based on previous stride analyses, greater speed is associated with fewer strides per 200 m track sectional and greater stride lengths^25, 30, 36, 37^. For higher speeds, the small reduction in the proportion of fatigue life due to fewer cycles of load is overwhelmed by the increase in fatigue accumulation due to the higher estimated load. The relationship between load and fatigue accumulation also explains the strong spatial association between areas of high load across a joint surface and subchondral bone injury^32^.

Horse level factors of age, sex and performance (as indicated by finishing position) influenced the percentage fatigue life accrued over each race start but in different ways; i.e. differences in numbers of strides, differences in speed or a combination of the two. Based on our model, females accumulate greater fatigue per race than male horses. This was due to the greater number of strides female horses took per race for similar speeds, and supported by a study of 2-year-olds galloping (under non-race conditions) where for any given speed, colts had longer stride length than fillies^30, 37^. There was a change in direction of relationship between horse sex and estimated fatigue life used in races in multivariable modelling compared to univariably, with geldings univariably using more fatigue life to females but less in multivariable analysis. This can be explained in part because a higher proportion of geldings competed in longer distance races (accounting for 61% of starters in races over 2000 to 2400 m and 72% of starters in races 2400 m and above), and geldings tended to carry heavier weights (56.41 ± 2.06 kg) compared to females (54.93 ± 1.73 kg). Greater horse age was associated with greater estimated bone fatigue per race. However, age was not associated with the number of strides, therefore the relationship demonstrated here is due to higher speeds observed in older horses^30^. Older racehorses tend to have higher stride frequencies which is consistent with higher speed if stride length is unchanged^30, 38, 39^. Average race speed has also been reported to increase with age until 4.5 years followed by a plateau period^40–42^. According to our previous investigation, the effect of finishing position on strides is variable depending on the stage of the race, but overall horses with better finishing positions take similar or slightly fewer strides than the rest of the field and average higher speed over race starts^30, 43^.

With increasing race distance total bone fatigue accumulation increased, but not at a proportional rate. This was because the number of strides per 200 m reduced, as did mean speed^30^. Horses racing on a softer surface consumed less bone fatigue life than those racing on firm surfaces, since the slower speed over-rides the effect of greater numbers of strides on softer surfaces^30^. Although a firm 2 track rating didn’t follow the pattern of greater fatigue life accrued than a good-rated track, only 0.08% of race starts (n = 20) were conducted on this surface type, therefore little weight can be placed on this finding. The stride characteristics of synthetic tracks (strides per 200 m and stride duration) were previously demonstrated to align with good rated turf tracks^30^, but the fatigue life estimate here aligned more with the soft track ratings, therefore a combination of speed and stride characteristics may be contributing to this effect. However, as there was only one racetrack with a synthetic surface in the dataset, other factors such as track size and undulation/changes in gradient may also have influenced the stride characteristics identified.

The relationship of weight carried on fatigue accrual was dependent on the class of race, where greater weight carried did not necessarily result in greater fatigue damage. Our previous investigation showed that overall the number of strides per race tended to decrease with increasing weight carried, and greater weight carried is reported to be associated with slower racing times^30, 42^. For the lower classed races, the estimated fatigue damage did not increase with greater weight carried due to a slight increase in speed compensating for fewer strides. A similar effect has been described in a Japanese cohort, where for young horses entering racing, speed increased despite additional weights carried^40^. Those authors speculated this was likely due to horse growth in the early racing years outweighing the effects of carried weight. Comparatively, the estimated fatigue accrual for open class races in this study increased with weight carried due to a greater increase in speed. Therefore, weight carried is an indicator of the ability of a horse as open class races tend to have the largest variation in horse ability. However, given the low number of starters carrying high weights, further investigation is required to fully assess the effect of heavy weights carried on fatigue accrual.

The results presented here largely agree with injury risk factor studies, especially for speed, race-level factors and horse age. This likely reflects the important contribution of speed and stride characteristics in injury development and supports the validity of the models presented. Race-factors of longer race distances and firmer turf track surfaces were associated with greater fatigue life, in agreement with risk factor studies in Australia^44, 45^. For track surface type, although some studies have reported a higher risk for specific fracture site locations on synthetic/all-weather tracks^46–50^, in pooled meta-analysis there was no difference in CMI risk for turf tracks compared to synthetic (*p* = 0.991)^6^. This is consistent with fatigue accumulation for synthetic tracks equivalent to the mid-range of turf track ratings. Better finishing position association with greater fatigue damage aligns with findings that horses expected to be better performers in race starts (as defined by better “Odds ranks”) are at higher risk of fracture^51^. Greater horse age was associated with greater estimated accumulated bone damage per race and similarly reported to be a risk factor for CMI in pooled meta-analysis^6^. Given that bone injuries are typically the result of damage accumulation over time, increased risk with age may be due to accumulated galloping distance. However, more fatigue damage may also accrue with each start in older horses. The lack of consistent association between weight carried and fatigue life is also interesting given that there is limited literature suggesting an association between weight carried and risk of injury. Of the 11 epidemiological studies in Thoroughbreds that have assessed the effect of weight carried on risk of CMI, 10 found no significant association^1, 6, 44, 52–60^, with only one study reporting greater carried weights to be a (univariable) risk factor^45^.

Where our results do not mimic injury risk, studies suggest areas where our modelling methods may be improved upon. In estimating fatigue accrual by horse sex, females used a greater percentage fatigue life over race starts. However males are typically higher risk of CMI^6^. Speeds achieved by mature horses have been shown to be not significantly different between males and females, but females undertake such speed at a greater stride frequency^37^. Stride characteristics in Thoroughbreds is a recent area of investigation, but to reduce injury risk in human athletics, it is preferential for athletes to increase their running cadence (stride frequency) and to reduce their stride length to reduce limb loading^61^. Our modelling will underestimate any protective effect of the higher stride frequency in female horses because the effect of stride frequency on limb load was not taken into account. Other aspects such as increased weight carried and horse weight will likely increase limb loading if speed is maintained due to a direct effect on the ground reaction force (GRF). Additional factors that are not considered with the current modelling method may contribute to these apparently conflicting results by affecting limb loads include muscle forces, bone shape and quality.

The current modelling approach would be improved by the addition of stance duration data (time that the hoof is in contact with the ground) as this would enable the measurement of duty factor (proportion of the stride the limb is in contact with the ground) from which GRF can be calculated^62^. We were also not able to account for potential differences in loading between individual limbs, for example whether or not a limb was leading during each stride which warrants consideration in future investigations. However, whilst higher GRFs have been identified in non-lead limbs during slow cantering^62^, the difference in load between lead and non-lead limbs reduces as speed increases, with no difference observed in GRFs or stance duration for galloping horses^25, 62^. The previously published equations we used do not have confidence intervals around their coefficients. Given the variation in strides between individual horses, the inclusion of a sensitivity analysis for vertical force estimates would account for further variation in these parameters. The coefficients for many of the presented variables are small, representing minor (often < 1%) differences in percentage fatigue life accrued between groups at the individual race start level. However, when tracked over a horses entire racing career these differences could result in substantial variation in the cumulative fatigue life accrued and therefore likelihood of bone failure. Future longitudinal investigations are therefore warranted. Future modelling would also benefit from the inclusion of speed and stride parameters incurred during training to enable investigate incurred load over a greater range of speeds and more accurately quantify horses’ total bone fatigue damage.

## Conclusions

The modelling approach described here builds on previous methods for determining racing loads by adding a bone fatigue function. The validity of this approach is demonstrated by the good agreement of the outputs with the results of previous risk factor studies, where factors such as greater horse age and better finishing positions, longer race distances and firmer track surfaces were associated with greater fatigue accumulation mimicking known risk factors for CMI, and has enabled identification of areas where modelling inputs can be improved. The results presented here suggest that there is substantial variation across horse- and race-level factors for fatigue accumulation in Thoroughbred racing. There is also substantial inter-horse variation in fatigue accumulation based on individual horses’ stride characteristics which explains why horses with similar racing histories may have very different outcomes.

## Supplementary Information

Supplementary Information.

## Data Availability

Data and associated materials are owned by proprietary bodies and therefore not available for distribution.

## List of symbols

*x*: Speed
σ: Joint load (MPa)
N_f_: Number of cycles to failure
FL_stride_: Proportion of fatigue life accrued per stride
FL_% race_: Percentage of fatigue life accrued per race

## References

[1] Cohen NC, Berry SM, Pelso JG, Mundy GD, Howard IC (2000). Association of high-speed exercise with racing injury in Thoroughbreds. J Am. Vet. Med. Assoc..

[2] Estberg L (1995). Cumulative racing-speed exercise distance cluster as a risk factor for fatal musculoskeletal injury in Thoroughbred racehorses in California. Prev. Vet. Med..

[3] Estberg L (1996). High-speed exercise history and catastrophic racing fracture in Thoroughbreds. Am. J. Vet. Res..

[4] Estberg L (1998). A case-crosover study of intensive racing and training schedules and risk of catastrophic musculoskeletal injury and lay-up in Californian Thoroughbred racehorses. Prev. Vet. Med..

[5] Hitchens PL, Hill AE, Stover SM (2018). Relationship between historical lameness, medication usage, surgery, and exercise with catastrophic musculoskeletal injury in racehorses. Front. Vet. Sci..

[6] Hitchens, P., Morrice-West, A. V., Stevenson, M. & Whitton, R. C. Meta-analysis of risk factors for racehorse catastrophic musculoskeletal injury in flat racing. *Vet. J.* **245** (2018). 10.1016/j.tvjl.2018.11.014 30819423

[7] Reed SR, Jackson BF, Wood JL, Price JS, Verheyen KL (2013). Exercise affects joint injury risk in young Thoroughbreds in training. Vet. J..

[8] Verheyen K, Price J, Lanyon L, Wood J (2006). Exercise distance and speed affect the risk of fracture in racehorses. Bone.

[9] Rogers CW, Firth EC (2004). Musculoskeletal responses of 2-year-old Thoroughbhred horses to early training. 2. Measurement error and effect of training stage on the Relationship between objective and subjective criteria of training workload. N. Z. Vet. J..

[10] Rogers CW (2007). Describing workload and scientific information on conditioning horses. Equine Comparat. Exercise Physiol..

[11] Stares J (2018). Identifying high risk loading conditions for in-season injury in elite Australian football players. J. Sci. Med. Sport.

[12] Cummins C (2019). Modelling the relationships between volume, intensity and injury-risk in professional rugby league players. J. Sci. Med. Sport..

[13] Bazzano M (2016). Application of a combined global positioning and heart rate monitoring system in jumper horses during an official competition—A preliminary study. Acta Vet. Hung..

[14] Fonseca RG, Kenny DA, Hill EW, Katz LM (2010). The association of various speed indices to training responses in Thoroughbred flat racehorses measured with a global positioning and heart rate monitoring system. Equine Vet. J..

[15] Vermeulen A, Evans DL (2006). Measurements of fitness in Thoroughbred racehorses using field studies of heart rate and velocity with a global positioning system. Equine Vet. J..

[16] Kingston J, Soppet GM, Rogers CW, Firth EC (2006). Use of a global positioning and heart rate monitoring system to assess training load in a group of thoroughbred racehorses. Equine Vet. J..

[17] Martig S, Chen W, Lee PV, Whitton RC (2014). Bone fatigue and its implications for injuries in racehorses. Equine Vet. J..

[18] Muir P (2008). Exercise-induced metacarpophalangeal joint adaptation in the Thoroughbred racehorse. J. Anat..

[19] Cui W (2002). A state-of-the-art review on fatigue life prediction methods for metal structures. J. Mar. Sci. Technol..

[20] Carter DR, Hayes WC (1977). Compact bone fatigue damage—I. Residual strength and stiffness. J. Biomech..

[21] Salkind, M. in *Composite Materials: Testing and Design (Second Conference)* 333–364 (ASTM International, 1972).

[22] Martig S, Lee PV, Anderson GA, Whitton RC (2013). Compressive fatigue life of subchondral bone of the metacarpal condyle in thoroughbred racehorses. Bone.

[23] Carter DR, Caler WE, Spengler DM, Frankel VH (1981). Uniaxial fatigue of human cortical bone. The influence of tissue physical characteristics. J. Biomech..

[24] Dendorfer S, Maier HJ, Taylor D, Hammer J (2008). Anisotropy of the fatigue behaviour of cancellous bone. J. Biomech..

[25] Witte TH, Hirst CV, Wilson AM (2006). Effect of speed on stride parameters in racehorses at gallop in field conditions. J. Exp. Biol..

[26] Parkin T (2006). Catastrophic fracture of the lateral condyle of the third metacarpus/metatarsus in UK racehorses–fracture descriptions and pre-existing pathology. Vet. J..

[27] Bailey CJ, Reid SWJ, Hodgson DR, Rose RJ (1999). Impact of injuries and disease on a cohort of two- and three-year-old thoroughbreds in training. Vet. Rec..

[28] Barr E, Pinchbeck G, Clegg P, Boyde A, Riggs C (2009). Post mortem evaluation of palmar osteochondral disease (traumatic osteochondrosis) of the metacarpo/metatarsophalangeal joint in Thoroughbred racehorses. Equine Vet. J..

[29] Harrison SM, Whitton RC, Kawcak CE, Stover SM, Pandy MG (2010). Relationship between muscle forces, joint loading and utilization of elastic strain energy in equine locomotion. J. Exp. Biol..

[30] Morrice-West AV (2021). Variation in GPS and accelerometer recorded velocity and stride parameters of galloping Thoroughbred horses. Equine Vet. J..

[31] Rubio-Martínez LM, Cruz AM, Gordon K, Hurtig MB (2008). Mechanical properties of subchondral bone in the distal aspect of third metacarpal bones from Thoroughbred racehorses. Am. J. Vet. Res..

[32] Harrison SM, Whitton RC, Kawcak CE, Stover SM, Pandy MG (2014). Evaluation of a subject-specific finite-element model of the equine metacarpophalangeal joint under physiological load. J. Biomech..

[33] Baum CF (2008). Stata tip 63: Modeling proportions. Stata J..

[34] Royston, P. BOXTID: Stata module to fit Box-Tidwell and exponential regression models (2013).

[35] Shaktivesh S, Malekipour F, Whitton RC, Hitchens PL, Lee PV (2020). Fatigue behavior of subchondral bone under simulated physiological loads of equine athletic training. J. Mech. Behav. Biomed. Mater..

[36] Ratzlaff M, Shindell R, White K (1985). The interrelationships of stride lengths and stride times to velocites of galloping horses. J. Equine Vet. Sci..

[37] Seder JA, Vickery CE (2003). Temporal and kinematic gait variables of Thoroughbred Racehorses at or near racing speeds. J. Equine Vet. Sci..

[38] Parkes RS, Weller R, Pfau T, Witte TH (2019). The effect of training on stride duration in a cohort of two-year-old and three-year-old Thoroughbred racehorses. Animals.

[39] Ferrari M, Pfau T, Wilson AM, Weller R (2009). The effect of training on stride parameters in a cohort of National Hunt racing Thoroughbreds: A preliminary study. Equine Vet. J..

[40] Takahashi T (2015). The effect of age on the racing speed of Thoroughbred racehorses. J. Equine Sci..

[41] Oki H, Willham R, Sasaki Y (1994). Genetics of racing performance in the Japanese Thoroughbred horse: I. Description of the data. J. Anim. Breed. Genet..

[42] Martin G, Strand E, Kearney M (1996). Use of statistical models to evaluate racing performance in Thoroughbreds. J. Am. Vet. Med. Assoc..

[43] Spence AJ, Thurman AS, Maher MJ, Wilson AM (2012). Speed, pacing strategy and aerodynamic drafting in Thoroughbred horse racing. Biol. Lett..

[44] Boden, L. A. *et al.* Risk factors for Thoroughbred racehorse fatality in flat starts in Victoria, Australia (1989–2004). *Equine Vet. J.* **39** (2007). 10.2746/042516407x183162 17910268

[45] Bailey CJ, Reid SWJ, Hodgson DR, Bourke JM, Rose RJ (1998). Flat, hurdle and steeple racing risk factors for musculoskeletal injury. Equine Vet. J..

[46] Williams RB, Harkins LS, Hammond CJ, Wood JLN (2001). Racehorse injuries, clinical problems and fatalities recorded on British racecourses from flat racing and National Hunt racing during 1996, 1997 and 1998. Equine Vet. J..

[47] Kristoffersen M, Parkin T, Singer E (2010). Catastrophic biaxial proximal sesamoid bone fractures in UK Thoroughbred races (1999–2004): Horse characteristics and racing history. Equine Vet. J..

[48] Reardon RJ, Boden L, Stirk AJ, Parkin TD (2014). Accuracy of distal limb fracture diagnosis at British racecourses 1999–2005. Vet. Rec..

[49] Parkin TD (2006). Catastrophic fracture of the lateral condyle of the third metacarpus/metatarsus in UK racehorses - fracture descriptions and pre-existing pathology. Vet. J..

[50] Parkin T (2004). Risk of fatal distal limb fractures among thoroughbreds involved in the five types of racing in the United Kingdom. Vet. Rec..

[51] Georgopoulos SP, Parkin TDH (2017). Risk factors for equine fractures in Thoroughbred flat racing in North America. Prev. Vet. Med..

[52] Bailey CJ, Reid SWJ, Hodgson DR, Suann CJ, Rose RJ (1997). Risk factors associated with musculoskeletal injuries in Australian Thoroughbred racehorses. Prev. Vet. Med..

[53] Bolwell C, Rogers C, Gee E, McIlwraith W (2017). Epidemiology of musculoskeletal injury during racing on New Zealand racetracks 2005–2011. Animals.

[54] Allen S, Rosanowski S, Stirk A, Verheyen K (2017). Description of veterinary events and risk factors for fatality in National Hunt flat racing Thoroughbreds in Great Britain (2000–2013). Equine Vet. J..

[55] Cohen N (1997). Racing-related factors and results of prerace physical inspection and their association with musculoskeletal injuries incurred in thoroughbreds during races. J. Am. Vet. Med. Assoc..

[56] Cohen ND, Mundy GD, Peloso JG, Carey VJ, Amend NK (1999). Results of physical inspection before races and race-related characteristics and their association with musckuloskeletal injuries in Thoroughbreds during races. J. Am. Vet. Med. Assoc..

[57] Henley WE, Rogers K, Harkins L (2006). A comparison of survival models for assessing risk of racehorse fatality. Prev. Vet. Med..

[58] Hernandez J, Hawkins DL, Scollay MC (2001). Race-start characteristics and risk of musculoskeletal injury in Thoroughbred racehorses. J. Am. Vet. Med. Assoc..

[59] Parkin T (2004). Horse-level risk factors for fatal distal limb fracture in racing Thoroughbreds in the UK. Equine Vet. J..

[60] Parkin T (2005). Risk factors for fatal lateral condylar fracture of the third metacarpus/metatarsus in UK racing. Equine Vet. J..

[61] Schubert AG, Kempf J, Heiderscheit BC (2014). Influence of stride frequency and length on running mechanics: A systematic review. Sports Health.

[62] Witte T, Knill K, Wilson A (2004). Determination of peak vertical ground reaction force from duty factor in the horse (Equus caballus). J. Exp. Biol..

