# Supplementary material for: Relationship between Thoroughbred workloads in racing and the fatigue life of equine subchondral bone
Source: Sci Rep. 2022 Jul 7;12:11528. doi: 10.1038/s41598-022-14274-y (PMC9262984; doi:10.1038/s41598-022-14274-y)
Supplement: Supplementary file 1 — Supplementary Information. [file 41598_2022_14274_MOESM1_ESM.docx]

**Supplementary Table S1. Univariable associations between horse and race-level factors on the percentage of fatigue life accrued over Thoroughbred race starts in Tasmania, Australia, according to estimated joint loads scaled to a maximum of 90 MPa. For categorical variables, the number and percentage of race starts in each category are presented.**

| **Race percentage fatigue life** | **Coef.** | **[95% Conf** | **Interval]** | **p-value** |
| --- | --- | --- | --- | --- |
| **Horse factors** |  |  |  |  |
| Sex |  |  |  |  |
| Female | (reference) |  |  |  |
| Gelding | 0.194 | 0.086 | 0.302 | <0.001 |
| Colt/Stallion | -0.167 | -0.478 | 0.144 | 0.291 |
| Age (years) | 0.297 | 0.264 | 0.329 | <0.001 |
| Finishing position | -0.033 | -0.042 | -0.025 | <0.001 |
| Weight carried (per 10 kg) | 0.025 | -0.154 | 0.203 | 0.270 |
|  |  |  |  |  |
| **Race Factors** |  |  |  |  |
| Race Distance ^±^ | 7.586 | 7.475 | 7.696 | <0.001 |
| Track type & Rating |  |  |  |  |
| Synthetic | (reference) |  |  |  |
| Firm 2 | -1.016 | -1.230 | -0.802 | <0.001 |
| Good 3 | 1.065 | 0.950 | 1.180 | <0.001 |
| Good 4 | 0.928 | 0.855 | 1.000 | <0.001 |
| Soft 5 | 0.547 | 0.469 | 0.625 | <0.001 |
| Soft 6 | -0.049 | -0.147 | 0.049 | 0.327 |
| Soft 7 | -0.099 | -0.212 | 0.014 | 0.086 |
| Heavy 8 | -0.411 | -0.502 | -0.320 | <0.001 |
| Heavy 9 | -0.749 | -0.885 | -.0614 | <0.001 |
| Heavy 10 | -1.546 | -1.727 | -1.365 | <0.001 |
| Race Class |  |  |  |  |
| Maiden/Class 1 | (reference) |  |  |  |
| Class 2-5 | -0.063 | -0.130 | 0.003 | 0.061 |
| Restricted (HCP/BM) | 1.043 | 0.948 | 1.139 | <0.001 |
| Listed /Group | 2.649 | 2.426 | 2.873 | <0.001 |
| Open | 0.346 | 0.177 | 0.515 | <0.001 |

^±^ Race distance scaled per 100 m and transformed to the power of 0.3531 according to a Box-Tidwell transformation
